# Supplementary material for: Information about confirmatory studies required for new drugs conditionally approved by Health Canada: A cross-sectional study
Source: PLoS One. 2022 Oct 20;17(10):e0276672. doi: 10.1371/journal.pone.0276672 (PMC9584409; doi:10.1371/journal.pone.0276672)
Supplement: Attachment — Submitted filename: Response to editor and reviewers.docx [file pone.0276672.s003.docx]

Dear Dr. Naudet:

Thank you and the reviewers for your comments. I have responded to each of the comments below and look forward to hearing from you.

Sincerely

Joel Lexchin MD

**Comments from the academic editor:**

I agree that more details are needed, especially concerning the methods and the results. Of course, you are expected to take all the comments from the 2 reviewers into consideration in your revised manuscript but please have a very strong focus on the methods and the results, in order to make sure that all important aspects for reproducibility purpose are present.

*The Methods and Results sections have been revised taking into consideration the comments from the reviewers.*

The use of one or more reporting guideline may help (see on the EQUATOR NETWORK). In case more material needs to be share, it can be attached as a supplement or posted on the Open Science Framework.

*The Strobe checklist is included in the submission of the revised manuscript.*

Please also make it explicit if a protocol was registered for this study (and where) and in case, no, please add a few words about that in the limitation section.

*A statement has been added to the limitations that there was no protocol registered in advance.*

Please also write a few words about the main limitation in the abstract to avoid any spin.

*The Discussion section in the Abstract now states “All of the data were gathered by a single individual possibly introducing unintended biases.”*

**Reviewer #1:**

Overall, the methodology of the study seems sound, but I had troubles understanding what is the specific definition of the sample of drugs that were analyzed and I had troubles to understand if the info that were analyzed were info about what the confirmatory studies were expected to be before completion, or info about how confirmatory studies were indeed conducted. I'm confident the confusion I've experienced is just a matter of clarification within the text and not due to design flaws, so I'm confident it will be easily resolved.

*I trust that my responses below will clarify any confusion that the reviewer had.*

However, currently, I was not able to fully understand what truly the findings of the study are about. I will try to pinpoint below why I've experienced this confusion. The study analyzes 78 drugs with NOC/c that were granted since the beginning of the program. It is specified it is divided into 39 drugs for which the condition of the program were "fulfilled", 34 were "unfulfilled" and 5 drugs were discontinued from the market before fulfilling their conditions. But what does "fulfilled" and "unfulfilled" really mean? At first, I was under the impression "fulfilled" means the confirmatory study was completed AND the drug was granted a full NOC. Thus, "unfulfilled" means the confirmatory study was completed AND the drug failed to obtain a full NOC. But, the data analysis section indicates "the median number of days from granting the NOC/c until fulfillment or until May 18, 2022 (date of database lock) for drugs that had not fulfilled their conditions was estimated". Therefore, does it means "fulfilled" only describes drugs for which confirmatory studies were completed whether the results were positive or not, and "unfulfilled" only describes drugs for which the confirmatory study is still under conduct? This aspect needs to be clarified for understanding what is the definition of the "population" analyzed in the study.

*In the Introduction unfulfilled is now defined as either the confirmatory studies not having been completed or being completed but not satisfactory to Health Canada and fulfilled is defined as completed and satisfactory to Health Canada.*

Of note: I have looked reference number 6 of the paper which indicates a sample of 89 new drugs and new indications with NOC/c until May 2017. I understand the present study does not analyze new indications, but still why only 78 drugs if this study covers a period until May 2022?

*Out of the 89 NOC/c analyzed in reference 6, 52 were for new drugs and 37 were for new indications of existing drugs. Therefore, between May 2017 and May 2022 there were an additional 26 NOC/c issued for new drugs. The number of NOC/c issued annually is variable and depends on manufacturers’ submissions.*

Moreover, I was confused about the nature of the info that are recorded under the Qualifying Notices policy. Does those info are only about what will be the confirmatory studies as provided by the health technology manufacturer when granted a NOC/c (therefore prospective info about what will be done) or is it retrospective info on how the confirmatory studies were indeed conducted, or is it a mix of both? Indeed, the study tackles data such as expected date of completion (therefore info about what the confirmatory study will be conducted), but also compares outcomes of the initial study leading to NOC/c and outcomes expected in confirmatory studies (the word expected seems to imply the info for confirmatory studies are info about what will be expected and not what has been done, but I was not sure which one it really was).

But, the study also tackles info about patient demographics and sample size. Here, I was under the impression the study is about info on how the confirmatory studies were really conducted and collected after completion. In the end, I think the paper needs to clarify what's what in order to be fully able to understand the results.

*The Introduction points out that the current study uses the perspective of clinicians in examining the information about the confirmatory studies in the QNs. Specifically, will these studies provide clinicians with knowledge that will help resolve uncertainties about the benefits of drugs with a NOC/c: a) what are the demographics of the patients who will be enrolled; b) what methodology will the studies employ (e.g., randomization and blinding); and c) if the drugs were given a NOC/c based on surrogate outcomes will the confirmatory studies use clinical outcomes. In addition, are two other pieces of information that are potentially important to clinicians in the QNs: when will the studies be completed so that they can take necessary precautions in prescribing until that point is there enough identifying information about the studies that will enable clinicians to search for and read the studies after they have been completed.*

I will now discuss more specific details.

INTRODUCTION

The date of the beginning of the NOC/c program could be clearly specified.

*The Introduction now states that the NOC/c pathway started in 1998.*

Page 5. After the first paragraph, I think the eligibility criteria a drug needs to fulfill for the NOC/c program could be specified in accordance with the Health Canada website in addition to the goal of the program.

*The Introduction now says that confirmatory studies are evaluated similar to any studies done before a drug is marketed and provides a reference to the relevant Health Canada document.*

Page 5. It is specified that before February 2003 QN did not exist and info were provided into a letter of understanding. Therefore, does it makes sense to analyze drugs since the begining of the NOC/c program instead of the begining of the request for a QN? Was it expected the same info were requested under the letter of understanding policy?

*This study evaluated all NOC/c issued since the start of the pathway. The Introduction now points out that the Letter of Undertaking needs to outline commitments for confirmatory studies that will be done in accordance with established scientific standards and the timelines for these studies that will verify the clinical benefit of the drug. Therefore, it is expected that the Letter of Undertaking should contain the same information as the information in the QN.*

Page 6. Please, specify why QNs for new indications were excluded from the study.

*QNs for new indications for existing drugs were not analyzed for a number of reasons: a) Health Canada does not provide the date when the application for the new indication was filed; b) there is no publicly available source about whether surrogate or clinical outcomes were used in granting a NOC/c for the new indication; and c) when a new indication is granted clinicians will have had some experience prescribing the drug and therefore the information in the confirmatory studies may be less important for them.*

METHODS

What was the criteria for deciding an outcome was a clinical or surrogate one?

*From January 1, 2005 documents on Health Canada’s Summary Basis of Decision website (*[*https://hpr-rps.hres.ca/reg-content/summary-basis-decision-result.php?lang=en&term=*](https://hpr-rps.hres.ca/reg-content/summary-basis-decision-result.php?lang=en&term=)*) have explained why the agency authorized drugs for sale in Canada. One of those documents describes the pivotal studies that were submitted and the primary outcomes in those studies. On the basis of the descriptions, the outcomes were classified as either surrogate or clinical and the type of outcome was recorded.*

On a related note, the method should clearly states data were only retrieved and analyze by one person, therefore there were no methods for reducing measurement biases such as inter-rater agreement.

*The Methods section already states that all of the information was gathered by a single person and the Limitations section repeats that information.*

RESULTS

In general, does "undetermined" characteristics (such as study outcome, randomization) corresponds to missing data, or data that were not reported with sufficient clarity for understanding the info, or a mix of both? I think the paper could benefit from a little more detail about this aspect.

*The term “undetermined” has been replaced by “unknown” and it is made clear that a result is unknown because of a lack of information.*

**Reviewer #2:**

The paper authored by Joel Lexchin throws a glance on regulatory decisions and requirements following conditional or accelerated approval of drugs in Canada. In a cursory approach, 78 conditional approval decisions by Health Canada were retrieved from the regulator’s website and other sources, which are not detailed.

*I am not sure why the reviewer considers this to be a cursory approach but if s/he could provide more details I would be happy to try and respond.*

Names of the specific drugs, year of conditional approval, or the manufacturer are not provided.

*This information, along with all the other information extracted is now provided in a supplementary file.*

This article does not contribute new insights to the scientific debate on how shifting clinically important efficacy and safety assessments from before to after authorization affects our ability to obtain an unbiased assessment of the risk-benefit ratio and safety of newly developed drugs (1-4).

*The type of analysis that the reviewer is proposing is certainly valid and one approach would be through a narrative review or preferably a systematic review. One step in understanding how regulator required post-market studies affect our understanding of safety and efficacy compared to what was learned from pre-market studies is knowing what type of information the regulators have required from the post-market studies, e.g., are the studies asking the correct questions, studying the right populations, using strong methodology. The current study is attempting to answer some of those questions.*

Meanwhile, there are many reports that post-authorisation studies often fail to deliver—lots of studies are never started, many take years longer than planned, and some fail to confirm pre-authorisation results. Evidence on relevant outcomes often remains inconclusive for several years, (5-7) and post-authorisation safety events are seen more frequently for drugs with expedited approval.(8) The sketchy findings presented by Lexchin suggest that Health Canada seems to accept that the manufacturers are not adhering to post-authorisation study requirements, and drugs are only rarely withdrawn, as was described before.(2)

*I agree with the reviewer that drugs with an expedited approval have more post-authorization safety events than drugs with a standard review. In fact, one of my previous studies demonstrates that (**L**e**x**c**h**i**n* *J**.* *P**o**s**t**-**m**a**r**k**e**t* *s**a**f**e**t**y* *w**a**r**n**i**n**g**s* *for drugs approved in Canada under the Notice of Compliance with conditions policy. British Journal of Clinical Pharmacology 2015;79:847-859.) I also agree with the reviewer that relatively few drugs are withdrawn because their confirmatory have failed to show efficacy. Again, that is a topic that I have previously explored (*L*exchin J. A comparison of the Food and Drug Administration’s and Health Canada’s regulatory decisions about failed confirmatory trials for oncology drugs: an observational study. Journal of Pharmaceutical Policy and Practice 2021;14:93.* [*https://doi.org/10.1186/s40545-021-00375-y*](https://doi.org/10.1186/s40545-021-00375-y)*.) Contrary to what the reviewer implies, my study does not suggest that Health Canada accepts that manufacturers are not adhering to post-authorization study requirements. That may well be the case, but that would require identifying and analyzing the post-market studies and comparing the information in them to what Health Canada had initially required. However, that task would be difficult for two reasons: as my study shows, knowing what Health Canada has required and identifying published versions of post-authorization studies would both be very difficult to accomplish because of the paucity of information that Health Canada provides in the QNs. I now identify those consequences of the lack of information in the Discussion.*

The paper has major limitations. Methods, results and conclusions are not well founded. It is unclear how drugs approved under the NOC/c policy were identified and how exactly the information on QNs was retrieved and compiled in which time period.

*The Methods section clearly states that drugs with a NOC/c were identified from one of three sources: the NOC/c website, a previous study of mine (Lexchin J. Notice of Compliance with conditions: a policy in limbo. Healthcare Policy. 2007;2:114-22) and a study by Law (Law M. The characteristics and fulfillment of conditional prescription drug approvals in Canada. Health Policy. 2014;116:154-61.) QNs were downloaded from the NOC/c website and if they were not available there then an Access to Information request was used. If drugs were approved with a NOC/c before QNs were being used then an Access to Information request was made for the relevant Letter of Undertaking. The Methods section now states that all of the information was gathered between May 18-21 except for the QNs and Letters of Undertaking that were previously acquired through Access to Information.*

The tables are not clear and uninformative to the reader, totals are missing. In table 1, the calculation of total study numbers is N=70 for “Outcome” and “Randomization” but 69 for “Blinding”.

*I thank the reviewer for pointing out this error which has now been corrected.*

A descriptive table of availability of main characteristics for each of the 78 QNs (e.g. number of studies, study identification, duration of conditional approval procedures, drug name and class, number, age and sex of participants, date of conditional approval, and outcome measures in NOCs and QN) is not provided.

*Suppementary File 1 now provides all the information that was used in the study.*

The result section is vague and does not attempt to look for potential associations, e.g. between missing information and drug class or duration of conditional approval.

*The reviewer seems to be asking for a regression analysis which would presumably use date of NOC/c, drug class and time until fulfillment of conditions as independent variables. However, there are multiple potential dependent variables. If the reviewer could specify which dependent variables s/he thinks are important I could explore the value of doing regression analysis.*

The conclusion to call for a regulatory template for “confirmatory studies” is weak. Does the author think that manufacturers would better adhere to regulatory templates than to QNs and why should they? Would regulators be more restrictive by using the templates instead the QNs and why should they? Rather, the integrity of accelerated/conditional approval should be discussed. Is there really a justification for curtailing or totally omitting Phase III trials? Take Covid-19 mRNA vaccines as an example: After a median of only 2 months follow-up, the pivotal Phase III studies were unblinded and placebo-group participants were offered vaccination, thereby introducing bias which may severely impair the comparability of vaccinated and non-vaccinated participants in the remaining observation time of the study. In light of the many unknowns of Covid-19 mRNA vaccine efficacy and safety, stopping the randomized trials at the time of conditional/ emergency approval violates basic principles of good medical practice. (9) A very recent independent analysis of adverse events summary tables submitted to the FDA for emergency use approval shows that the risk of severe adverse events of special interest after mRNA vaccination exceeds the benefit risk reduction of hospitalization due to Covid-19. The authors call for a “systematic review and meta-analysis using individual participant to address questions of harm-benefit in various demographic subgroups. Full transparency of the COVID-19 vaccine clinical trial data is needed to properly evaluate these questions. Unfortunately, well over a year after widespread use of COVID-19 vaccines, participant level data remain inaccessible.” (10) Therefore, my conclusion is to omit conditional approval wherever possible. Access to Individual participant data (IPD) should be granted for independent scrutiny for all drug approval pathways.

*As per my answer to one of the reviewer’s previous comments, I agree that it would be important to have a much stronger understanding of what Health Canada requires in confirmatory studies, whether those requirements will provide the necessary information to resolve questions about benefits and whether manufacturers are adhering to those requirements. Therefore, as a first step in this process I think that a call for Health Canada to develop a template of what information should be included in the QNs about confirmatory studies is a reasonable requirement. Once that step is taken, then we can start to answer the questions posed above about confirmatory studies in particular and the NOC/c pathway in general.*

*The reviewer uses the problems with the Phase III study of the Pfizer COVID vaccine to argue against omitting pre-market Phase III studies which is what the NOC/c process does. Again, I agree with the reviewer that omitting Phase III studies often leads to an overestimating of the benefits of drugs and an underestimation of harms as the FDA report “22 case studies where phase 2 and phase 3 trials had divergent results”. Arguing for the elimination of NOC/c and similar pathways is valid but that was not the purpose of my study.*
