# Supplementary material for: Information about confirmatory studies required for new drugs conditionally approved by Health Canada: A cross-sectional study
Source: PLoS One. 2022 Oct 20;17(10):e0276672. doi: 10.1371/journal.pone.0276672 (PMC9584409; doi:10.1371/journal.pone.0276672)
Supplement: Attachment — Submitted filename: Response to reviewers.docx [file pone.0276672.s004.docx]

I want to thank the academic editor for his careful and thorough management of my submission and the reviewers for their insightful comments. Below I indicate how I have responded to the individual comments.

**Reviewer #1:**

Overall, the revised version of the draft has alleviated the issues that I have raised in my previous review.

*I am glad that my revisions have met the reviewer’s standards.*

Minor remarks:

Introduction: I suggest to reword the sentence "Most approvals are based on promising surrogate outcomes" by "Most approvals are based on surrogate outcomes assumed to be validated for surrogacy". Indeed, it is discussed later that the validity of numerous surrogate outcomes can be doubtful. However, the current version of the sentence seems to imply all surrogate outcomes are promising.

*I have added a direct quotation from the Health Canada guidance document on its Notice of Compliance with conditions policy and the sentence now reads “Most approvals are based on promising surrogate outcomes that “are reasonably likely, based on available evidence, to predict an effect of a drug on recognized clinical outcomes such as morbidity and mortality.”*

Results section: There are still occurence within the manuscript and tables of the term "undetermined" instead of "unknown".

*All instances of the word “undetermined” have been deleted.*

Discussion page 17: There may be a typo in the sentence "whereas 14 were being tested in studies with further surrogate outcomes". Should it be 44 instead of 14?

*14 is the correct number but to avoid confusion I have added that the type of outcome in 23 cases was not specified.*

Conclusion: The new beginning of the conclusion could be modified a little to be more accurate regarding the findings. Indeed, the findings are striking regarding the lack of information in the QNs, but maybe the conclusion is not as strong as "virtually no one... has any knowledge...." because sometimes there are information.

*The start of the sentence now reads “That means that in most cases clinicians have little knowledge…”*

**Reviewer #2:** Thank you for revising the manuscript and addressing critical points, particularly providing the supplementary table with key information on the 78 drugs conditionally approved between 1998 and 2022 . From this table follows that:

1. The -use of conditional approval pathways is increasing. There were only 34 NOCs issued during the first 17 years between 1998 and 2014, i.e. roughly 2 per year. In contrast, between 2015 and 2021, 43 drugs were conditionally approved, about 6 drugs per year.

2. Conditionally approved drugs are increasingly focusing on lucrative cancer treatment. From 1998 through 2014 there were 6 NOC/c on drugs for HIV treatment, 19 for cancer, and 9 for the treatment of mixed conditions. During 2015-2021, all 43 except one were oncology drugs.

*The reviewer has pointed out some very interesting trends in the use of the NOC/c pathway*

*and I have previously published about these changes (up to the end of 2017), please see:*

*Lexchin J.* *Health Canada’s use of its Notice of Compliance with Conditions drug approval*

*policy: a retrospective cohort analysis.* *International Journal of Health Services 2019:49:294*

*305.*

3. Drug withdrawals in conditionally approved pathways decrease. Four of 34 conditionally approved drugs between 1998 and 2014 were withdrawn, among them the prominent example natrecor ®, an expensive „innovative“ drug for acute congestive heart failure which was shown to be a killer of patients by independent scientific evaluation of primary trial reports to the FDA and from the manufacturer (Sackner-Bernstein JD et al. JAMA 2005;293:1900-1905). After 2015, only one drug out of 43 was withdrawn by the manufacturer.

*I did not have the resources to do an extensive search for the reasons why drugs were withdrawn but I did search the NOC/c website to see what was recorded for these drugs. There was no information about 3 of them and for one the reason recorded was that the confirmatory studies did not demonstrate efficacy. This information is now mentioned in the text and I have added a column to Supplementary File 1 with this information.*

From the perspective of clinicians, it would be more informative to describe which 8 drugs had missing QNs, which drugs or drug class had no information on outcome (38), randomization (33) and blinding (39), for which drugs or drug class the number of patients and their ages (23) and surrogate outcomes were available (52) and for which drugs or drug class outcomes could not be determined (30).

*After further searching I was able to find 5 of the 8 missing QNs and have now included the information in them in the text and tables. (The inclusion of this additional information meant that many of the figures had to be changed.) In addition, I have now clarified in the text that all information was redacted from one QN and in the remaining two QNs the wrong documents were sent in response to the Access to Information request.*

*Interested readers can find the information about which drugs contained missing information in the QNs from Supplementary File 1. It is possible that there was some pattern to the exclusion of information but it is much more likely that the exclusion was the result of the inconsistency in how Health Canada constructed these documents. This inconsistency can also be found in the information in Summary Basis of Decision documents and in decisions about which drugs qualify for a NOC/c. These inconsistencies are now noted at the end of the first paragraph of the Discussion.*

According to the author, the aim of the paper is “to resolve uncertainties about the benefits of drugs with a NOC/c”. I do not see how the proposed templates can basically improve the sobering reality of conditional/expedited approval pathways. The rationale for omitting phase III pivotal trials i.e., to provide innovative treatments faster to patients with life-threatening illnesses must be questioned. Innovation does not automatically imply more good than harm. The conditional approval pathway puts patients, doctors, and society at high risks of no or marginal benefit and severe undiscovered adverse events at extremely high costs. In my opinion, scientists have to speak up against this bad clinical practice jointly committed by regulators and manufacturers.

*With respect to the reviewer, the aim of the study was not “to resolve uncertainties about the benefits of drugs with a NOC/c”. That would require examining the information in completed confirmatory studies and then determining if that information was sufficient to more definitively determine benefits and harms in the populations that were using the drugs. The aim of my study as stated in the Introduction was to examine the quantity and quality of the information in the Qualifying Notices.*

*As I said in my previous response to the reviewer, I agree that omitting Phase III studies often leads to an overestimating of the benefits of drugs and an underestimation of harms as the FDA report “22 case studies where phase 2 and phase 3 trials had divergent results” shows. However, I think that it is necessary to have a strong evidentiary basis to argue for the retention of Phase III studies in the drug approval process. Part of that evidentiary basis is having a much stronger understanding of the limitations of the NOC/c pathway. One aspect of that understanding is an examination of the requirements for confirmatory studies and whether those requirements will provide the necessary information to resolve questions about the benefits of drugs approved and whether manufacturers are adhering to those requirements. A first step in this process is a call for Health Canada to develop a template of what information should be included in the QNs. Once that step is taken, then we can start to answer questions posed about the value of confirmatory studies in particular and the NOC/c pathway in general.*
