# Supplementary material for: Information about confirmatory studies required for new drugs conditionally approved by Health Canada: A cross-sectional study
Source: PLoS One. 2022 Oct 20;17(10):e0276672. doi: 10.1371/journal.pone.0276672 (PMC9584409; doi:10.1371/journal.pone.0276672)
Supplement: Attachment — Submitted filename: Response to reviewer.docx [file pone.0276672.s005.docx]

I am grateful to the reviewer for evaluating the manuscript so rapidly. Below I explain how I have dealt with the comments.

**Reviewer #3:** Thank you for the opportunity to review this manuscript on the information about confirmatory studies required for new drugs conditionally approved by Health Canada. Overall, this manuscript is methodologically sound, easy to read and understand, and suitable for publication. However, I have a few comments that will further increase clarity.

*I thank the reviewer for her/his comments and am happy to hear that s/he felt that the manuscript was suitable for publication.*

Abstract Methods

“A list of drugs approved under the NOC/c policy and their QNs were sourced” .. please report from where?

*The following has been added to the end of the sentence “from two previous publications as well as Health Canada’s NOC/c website”.*

Abstract Results

- Provide time-frame for 78 drugs approved using NOC/c (dates)

*The following was added “from the time the first drug was approved under the program in July 1998 until May 18, 2022”.*

- Provide median number of studies per drug with IQR

*The following was added “median of 2 studies per QN, interquartile range 1,3”.*

- Should say proportion of men and women?

*The abstract already states that “No study gave the distribution of men and women”*.

- Where is information about other aspects of methodology that are considered primary endpoints?

*The following was added to the end of the Results section “**Specifically for oncology drugs, 44 were approved with surrogate outcomes and one with a clinical outcome. Eight (18.2%) of the 44 oncology drugs approved with surrogate outcomes had confirmatory studies that used clinical outcomes, 14 (31.8%) used surrogate outcomes and the outcome could not be determined for 22 (50.0%). The sole oncology drug approved with a clinical outcome had a confirmatory study with a surrogate outcome”.*

Abstract Discussion

- “Confirmatory studies with surrogate outcomes were often used to validate efficacy in drugs initially approved using surrogate outcomes” – often may not be the right word, considering it is <30%.

*“Often” was replaced by “almost one-third of the time”*.

Introduction

- Missing reference for “provide patients suffering from serious….”

*Reference 2 applies to this statement and has been added.*

- The author mentions that prior to 2/2003 the QN did not exist? The abstract and results section of the manuscript can be improved by more transparent reporting of dates

*The start of the first sentence in the Methods now reads “The generic name of all new drugs approved with a NOC/c along with the dates of granting and fulfillment of the NOC/c from the time that the first drug was approved under the program in July 1998 until May 18, 2022…”.*

*As indicated above, the same information is now included in the Results section of the Abstract.*

*I am not sure what other dates the reviewer would like me to include but I would be happy to add them if the reviewer can be more specific.*

- Some of the objectives in the introduction are not discussed in the abstract – when will the studies be completed and is there enough information about the studies that will enable them to search for and read the studies

*The following sentence was added to the Results section of the Abstract “The expected time of completion of the studies was available for 36 out of 154 and information to identify studies was present for 77, absent for 23 and unclear for 26.”*

- The final paragraph of the introduction seems like a methods section paragraph.

*The paragraph that the reviewer refers to has been shifted to the end of the “Information Extraction” subsection of the Methods.*

Results

- The reporting of characteristics at the QN level is rather confusing. I think the following reporting system could improve clarity:
  - At least one study randomized vs. no studies randomized
  - At least one study blinded vs. no studies blinded
  - At least one clinical outcome vs. no clinical outcomes

*The type of reporting suggested by the reviewer seems to assume that there is a binary result, e.g., yes or no, but there are multiple results for whether there was randomization, blinding and type of outcome. If the reviewer has a suggestion for a way to report multiple results, I would be happy to reconsider rewording.*

- Table 1 is a little difficult to follow. Please see suggestion above.

*The suggestion above seems to be for the text not for the table, but percentages have been added to the numbers in the table and in the text.*

Methodology in individual studies

- Please report %’s (e.g., 99 (X%)) for the numerators

*Percentages have now been added.*

- Table 2, please report %’s

*Percentages have been added to the table and the text.*

Patient demographics

- What about race/ethnicity or age?

*There was no information reported about race/ethnicity for any study and that information has been added in the Results. The Results already report information about age: “**The age of patients was given in 24 (15.6%) studies (1 study said that there should be a subpopulation aged greater than 65), but in 15 (9.7%) cases relatively vague terms such as “children”, “pediatric”, “adolescent” and “adult” were used.”*

- Other information

*The limitations subsection already says “The QNs contained other types of information, for example about the frequency of reporting of safety issues, and some QNs contained detailed descriptions of how drugs should be studied in confirmatory studies, but that information was not examined.”*

- Please report %’s

*Percentages have been added to all the numbers.*

Discussion

- In the opening of the discussion, please recap study design, time, and sample. E.g.. In this cross-section study of XXXXX…..between XXX and XXXXX

*The opening part of the first sentence of the Discussion now reads “This cross-sectional study of 75 Qualifying Notices issued by Health Canada for drugs approved through its NOC/c pathway from July 1998 to May 18, 2022 found that the QNs…”*

- The opening of the discussion highlights an outcome that was not included in the abstract and therefore does not appear to be a major finding – the information to identify studies.

*The following information has been added to the Results in the Abstract “information to identify studies was present for 77, absent for 23 and unclear for 26”.*

- What are SBD documents?

*SBD documents are already described in the subsection of the Methods “**Study outcome used in granting a NOC/c”.*

- The author could consider referencing [https://www.bmj.com/content/361/bmj.k2031](https://urldefense.com/v3/__https://www.bmj.com/content/361/bmj.k2031__;!!CjcC7IQ!N4_XjJCPpoKt8divFpOquWQis0aotih7_Ri-QxmB-1HCNQPCq4RLkodo7kLN9jiU1q9nzDH-07OeVb0qoNE$) , which focuses on the lack of information including in descriptions of postmarketing requirements for FDA-approved drugs. (disclosure: I am an author on this manuscript, so please do not feel any pressure to reference this paper. I just thought it had some similar and interesting findings).

*I am grateful to the reviewer for drawing this paper to my attention. I have cited it in the Discussion.*
